# Head-to-head comparison of biparametric versus multiparametric MRI of the prostate before robot-assisted transperineal fusion prostate biopsy

**DOI:** 10.1007/s00345-022-04120-1

**Published:** 2022-08-04

**Authors:** Wolfgang M. Thaiss, Simone Moser, Tobias Hepp, Stephan Kruck, Steffen Rausch, Marcus Scharpf, Konstantin Nikolaou, Arnulf Stenzl, Jens Bedke, Sascha Kaufmann

**Affiliations:** 1 Department of Diagnostic and Interventional Radiology, Eberhard-Karls-University, Hoppe-Seyler-Str.3, 72076 Tübingen, Germany; 2 Department of Nuclear Medicine, University Hospital Ulm, Albert-Einstein-Allee 23, 89081 Ulm, Germany; 3 Department of Urology, Siloah St. Trudpert Klinikum, Wilferdinger Str. 67, 75179 Pforzheim, Germany; 4 Department of Urology, Eberhard-Karls-University, Hoppe-Seyler-Str.3, 72076 Tübingen, Germany; 5 Department of Pathology and Neuropathology, Eberhard-Karls-University, Liebermeisterstr. 8, 72076 Tübingen, Germany; 6 Diagnostic and Interventional Radiology, Siloah St. Trudpert Klinikum, Pforzheim, Germany

**Keywords:** Magnetic resonance imaging, Image-guided biopsy, Prostate cancer, Dynamic contrasted-enhanced imaging, Prostate imaging reporting and data system, PI-RADS v2.1

## Abstract

**Purpose:**

Prostate biparametric magnetic resonance imaging (bpMRI) including T2-weighted imaging (T2WI) and diffusion-weighted imaging (DWI) might be an alternative to multiparametric MRI (mpMRI, including dynamic contrast imaging, DCE) to detect and guide targeted biopsy in patients with suspected prostate cancer (PCa). However, there is no upgrading peripheral zone PI-RADS 3 to PI-RADS 4 without DCE in bpMRI. The aim of this study was to evaluate bpMRI against mpMRI in biopsy-naïve men with elevated prostate-specific antigen (PSA) scheduled for robot-assisted-transperineal fusion-prostate biopsy (RA-TB).

**Methods:**

Retrospective single-center-study of 563 biopsy-naïve men (from 01/2015 to 09/2018, mean PSA 9.7 ± 6.5 ng/mL) with PI-RADSv2.1 conform mpMRI at 3 T before RA-TB. Clinically significant prostate cancer (csPCa) was defined as ISUP grade ≥ 2 in any core. Two experienced readers independently evaluated images according to PI-RADSv2.1 criteria (separate readings for bpMRI and mpMRI sequences, 6-month interval). Reference standard was histology from RA-TB.

**Results:**

PI-RADS 2 was scored in 5.1% of cases (3.4% cancer/3.4% csPCa), PI-RADS 3 in 16.9% (32.6%/3.2%), PI-RADS 4 in 57.6% (66.1%/58.3%) and PI-RADS 5 in 20.4% of cases (79.1%/74.8%). For mpMRI/bpMRI test comparison, sensitivity was 99.0%/97.1% (*p* < 0.001), specificity 47.5%/61.2% (*p* < 0.001), PPV 69.5%/75.1% (*p* < 0.001) and NPV 97.6%/94.6% (n.s.). csPCa was considered gold standard. 35 cases without cancer were upgraded to PI-RADS 4 (mpMRI) and six PI-RADS 3 cases with csPCa were not upgraded (bpMRI).

**Conclusion:**

In patients planned for RA-TB with elevated PSA and clinical suspicion for PCa, specificity was higher in bpMRI vs. mpMRI, which could solve constrains regarding time and contrast agent.

## Introduction

Prostate cancer (PCa) represents the most common non-cutaneous cancer among men with a lifetime risk of up to 37% [1]. Multiparametric magnetic resonance imaging (mpMRI) is regarded as the best imaging modality for the prostate and further standardization for describing and interpreting imaging results due to the introduction of the Prostate Imaging–Reporting and Data System (PI-RADS) and helps increase cancer detection based on the improved information on tissue characteristics [2]. Currently, PI-RADSv2.1 conform mpMRI demands T2-weighted sequences (T2w), diffusion weighted Imaging (DWI), and dynamic contrast-enhanced imaging (DCE). It is notable, however, that in PI-RADSv2.1 DCE-imaging is solely a secondary sequence for lesions in the peripheral zone (PZ) to further characterize lesions of the PI-RADS 3 category and not included in evaluation of lesions in the transition zone (TZ). The benefits of a biparametric MRI protocol (bpMRI) are signified by reduced costs, shorter acquisition time, and no contrast agent associated risks.

In general, the ability to differentiate PCa from focal benign processes like chronic prostatitis can be difficult. On MRI, both T2w and ADC derived from DWI can display decreased signal intensity in the PZ. The classic shape in form of a band or wedge is sometimes replaced by focal or irregular appearances. Additionally, also inflammatory processes can lead to increased DCE values by increased perfusion. Furthermore, PCa and chronic prostatitis are often accompanied by elevation of serum prostate-specific antigen (PSA) [3–5]. Especially in case of PI-RADS 3 lesions, histopathology reveals chronic prostatitis in about 50% [4, 6]. However, the value of DCE in the detection of prostate cancer is still controversial. Some studies have shown that combining DCE MRI with T2w and DWI does not significantly improve the diagnostic accuracy for prostate cancer [7–9].

Contrarily, some studies have found that DCE MRI is highly sensitive in the diagnosis of PCa [10–12], especially in peripheral lesions, and combining DCE MRI with DWI can significantly improve the accuracy of cancer detection. With the introduction of PI-RADSv2.0, the role of DCE MRI was limited to upgrade lesions from PI-RADS 3 to PI-RADS 4 in the peripheral zone when contrast enhancement is observed in these lesions.

In view of the potential advantages, it may be questionable whether dynamic contrast enhancement should be mandatory in routine prostate MRI protocols or if bpMRI provides similar results to those of mpMRI for the detection and localization of PCa.

The aim of our study was to compare the detection rate of PCa and csPCa of bpMRI on Prostate Imaging Reporting and Data System (PI-RADS) v2.1 scoring in comparison to the mpMRI approach in biopsy-naïve men with elevated prostate-specific antigen (PSA). Histology of targeted and systemic biopsy of mpMRI guided robot-assisted transperineal fusion prostate biopsy (RA-TB) was defined as the reference standard.

## Materials and methods

This retrospective study was approved by the institutional review board (359/2019BO2) and conducted in accordance with the Helsinki protocol. From January 2015 to September 2018, we included consecutive patients meeting the following inclusion criteria: rising and/or persistently elevated PSA, clinically indicated mpMRI of the prostate and planned for RA-TB without prior biopsy. Exclusion criteria were a PI-RADS score of 1 as they did not show a measurable or targetable lesion and/or a palpable tumor at digital rectal examination, as these were referred to biopsy without prior MRI. Digital rectal examination was performed by experienced urologist consultants (S.K., S.R., J.B.). Mean patient age was 66 ± 8 years (range 45–84 years).

### MR imaging

All patients underwent mpMRI on a 3 T MRI system according to the European Society of Urogenital Radiology guidelines and adapting the ACR Prostate Imaging–Reporting and Data System (PI-RADS) v2.1 guidelines. The MRI acquisition protocol was in accordance with the technical requirements stated in the PI-RADS v2.1 update. No endorectal coil was used. All patients received body weight adapted gadolinium-based intravenous contrast agent followed by a saline flush. All Patients received 20 mg hyoscine butylbromide i.v. before the examination.

Two board-certified uroradiologists with 5 and 12 years of experience in prostate MRI reading analyzed the acquisitions for both mpMRI and bpMRI. Readers were blinded to the histopathologic diagnosis as well as the clinical procedures after the MRI. There were 6 months in between reading sessions for mpMRI and bpMRI.

An index lesion was defined before biopsy, this was considered as “target” in contrast to “off-target” biopsy, which is equivalently used for systematic, non-targeted biopsy of the prostate.

### RI reader performance

MRI reader performance concordance analysis for PI-RADSv2.1 score was performed between two specialists for mpMRI. The agreement for both readers was substantial for both mpMRI (Kohen’s *κ* = 0.69, *z* = 8.05, *p* < 0.001) and bpMRI Kohen’s *κ* = 0.62, *z* = 9.75, *p* < 0.001). Importantly, for the PI-RADS 3 cases with upgrade to PI-RADS 4 in mpMRI, no reader differences were observed.

### Robot-assisted mpMRI-TRUS fusion prostate biopsy (RA-TB)

Biopsy was performed using an iSRobot Mona LisaTM robot unit, an ultrasound machine (Pro Focus 2202, BK Medical, Peabody, MA) with multi-frequency ultrasound probe (BK 8848, BK Medical, Peabody, MA) and UroBiopsyTM 3D modelling software (both: Biobot Surgical, Singapore) as previously reported [13]. Four targeted biopsy samples and 14 off-target transperineal biopsy samples were obtained with focus on the peripheral zones. All procedures were performed by an experienced urologist consultant. Tissue samples were fixated with formalin solution and evaluated for histology. Clinically significant prostate cancer (csPCa) was defined was defined as ISUP grade ≥ 2 in any core.

### Statistical analysis

Variables are presented as mean and standard deviation, confidence intervals (CI) are given when indicated. Group comparisons were calculated with Kruskal–Wallis ANOVA as parameters did not show normal distribution (Kolmogorov–Smirnov test) and were corrected for multiple comparison with Dunn's multiple comparisons test. GraphPad Prism version 9.0.0, GraphPad Software, San Diego, California, USA, was used for statistical analysis, significance was considered as *p* < 0.05. For calculation of the test accuracy of bpMRI and mpMRI and the statistical comparison between both tests, we used the “Compbdt” package for R (R Core Team (2021). R: A language and environment for statistical computing. R Foundation for Statistical Computing, Vienna, Austria. URL https://www.R-project.org/) as described and published previously [14]. Results of sensitivity and specificity were compared using the Wald test statistic to an alpha error of 5% and two-tailed McNemar test with Holm correction. Results for positive and negative predictive value (PPV, NPV) were compared using the Wald test statistic to an alpha error of 5% and weighted generalized score (WGS) test statistic.

## Results

### Patient characteristics

A total of 563 patients with mpMRI before RA-TB were included in this study. PSA value at time of MRI was 9.8 ± 6.4 ng/mL (range 1.2–39.0 ng/mL, 63.2% below PSA 10 ng/mL). No significant differences were found between PI-RADS groups for PSA density, except between PI-RADS 5 and PI-RADS 3 (mean rank difference 66.3, *H* = 11.8, *p* = 0.02).

### Biopsy results

337 of 563 patients (59.9%) showed cancer in at least 1 biopsy core from 18 cores. 321 of 337 patients were positive at the site of the target lesion. 16 carcinomas were found off-target only. 68 cases were positive only in the target biopsy but not in systemic biopsy. In 253 cases, both targeted biopsy and off-target biopsy were positive. In 230 of 337 tumors, the target biopsy showed the highest Gleason score compared to 107 cases with highest Gleason score in off-target biopsy, 14 of those 107 cases resulted in an upgrade to a csPCa while targeted biopsy showed a Gleason 6 score.

**Fig. 1:**
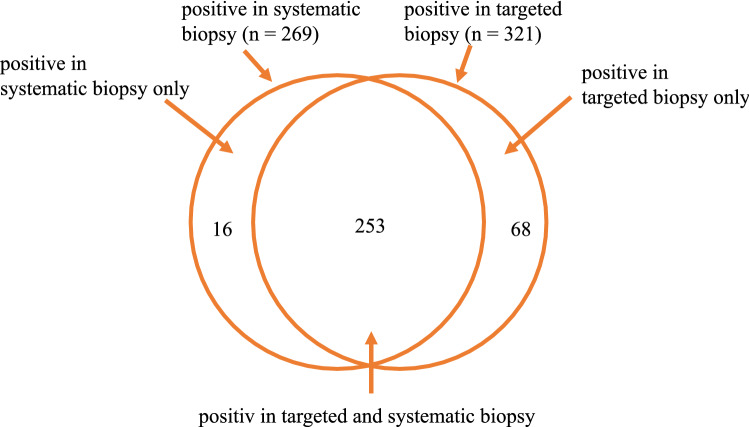
Venn diagram of positive biopsy results for prostate cancer (*n* = 337) indicating positive findings in targeted and systematic biopsy

Seven cases of PI-RADS 3 findings (all TZ) and eight cases of PI-RADS 4 cases (2 TZ, 6 PZ, none of them with contrast enhancement) were positive off-target only. From those, three cases with PI-RADS 3 showed csPCa, while none of the PI-RADS 4 cases was a csPCa. An overview of the biopsy results is provided in Fig. 1.

No severe complications, defined as Clavien–Dindo > Grade I were observed after biopsy, in 65 cases mild complications with transient urinary retention were documented.

### MRI results

The mean prostate volume (Table 1) derived from MRI measurements was 45 ± 22 ml, resulting in a PSA density of 0.25 ± 0.19, with 199 patients ≤ 0.15 and 364 patients ≥ 0.15. 255 of the later were diagnosed with cancer on RA-TB (243 with csPCa). Lesion size was 12 ± 5 mm (range 3 – 30 mm) with a lesion volume of 3.2 ± 2.5 cc (range 0.6–10.3 cc). Most lesions were found in the PZ (426) followed by the TZ (137). Time from MRI to biopsy was 33 ± 25 days (range 1 – 114).

**Table 1:** Characteristics of patients with final pathology

Parameter	*n* = 563	
Prostate volume (ml ± SD)	45 ± 22	
PSA level (ng/ml)		
≥ 20	43 (7.6%)	
10–20	153 (27.2%)	
≤ 10	367 (65.2%)	
PSA density mean ± SD	0.25 ± 0.19	
PSA density ≤ 0.15	199	
PSA density ≥ 0.15	364	

Histology	number	%
No cancer	226	40.1
Cancer	337	59.9
cnsPCa (Gleason score = 6)	49	
csPCa (Gleason score ≥ 3 + 4	288	
Gleason score 3 + 4	94	
Gleason score 4 + 3	72	
Gleason score 8	83	
Gleason score 9	32	
Gleason score 10	7	

c(n)sPCa clinically (not) significant prostate cancer, *SD* standard deviation

**Fig. 2:**
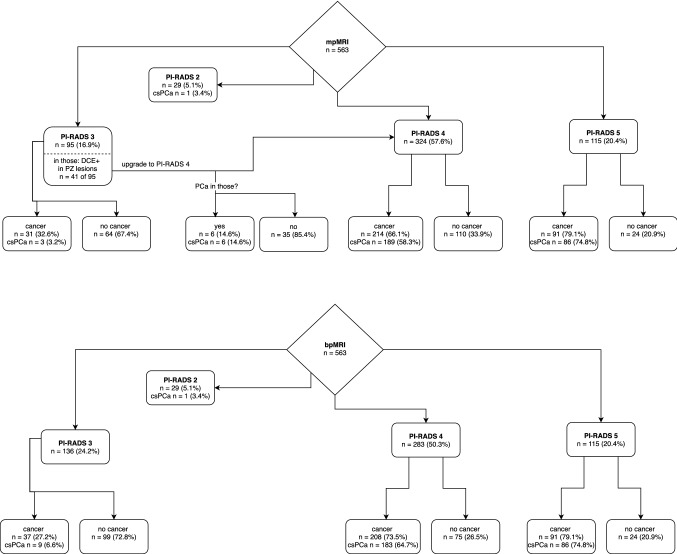
Flowchart for mpMRI reading (top) and bpMRI reading (bottom) with number and percentage of PI-RADS scores. Presence of cancer and number of clinically significant prostate cancer (csPCa) are outlined. PI-RADS 3 lesions located in the peripheral zone (PZ) that were positive in dynamic contrast-enhanced imaging (DCE +) are delineated

Flow charts for the results of mpMRI and bpMRI reading are summarized in Fig. 2. 29 out of 563 cases were classified as PI-RADS 2 (5.1%). Off-targeted biopsy was positive in one patient with non-suspicious mpMRI (PI-RADS 2, PSA 5.1 ng/ml, PSA density 0.2 ng/ml, 2/18 positive cores, Gleason score 4 + 3), resulting in a csPCa. A PI-RADS 5 score was given in 115 patients (20.4%). Of those, carcinomas were detected in 79.1% (91 out of 115). Here, 94.5% were classified as csPCa.

### mpMRI and bpMRI results

95 lesions (16.9% of all lesions) were scored as PI-RADS 3 in mpMRI reading. 31 of those cases were cancer-positive after biopsy (32.6%) with 3 cases of csPCa (3.2%).

PI-RADS 4 was selected in 324 (57.6%) cases with biopsy-proven carcinoma in 214 occasions (66.1%), 189 (58.3%) of those with csPCa.

41 cases were upgraded from PI-RADS 3 to PI-RADS 4 due to contrast enhancement of PZ lesions in mpMRI. Of those, 6 cases were cancer-positive on biopsy, all of them with a csPCa. Thirty-five lesions resulted in benign histology, such as prostatitis.

In bpMRI reading, 136 lesions (24.2% of all lesions) were scored as PI-RADS 3. 37 of those cases were cancer-positive after biopsy (27.2%) with 9 cases of csPCa (6.6%). PIRADS 4 was selected in 283 (50.3%) cases, biopsy-proven carcinoma in 208 occasions (73.5%), csPCa in 183 (64.7%).

### Performance of bpMRI and mpMRI

As no contrast agent was applied, six PI-RADS 3 lesions in the PZ were not upgraded to PI-RADS 4 in bpMRI. All of them were csPCa. With mpMRI, 35 cases were upgraded to PI-RADS 4 due to positive DCE with no proof of PCa in histology (10.8% of all PI-RADS 4 cases, Fig. 1).

Taken together, these findings resulted in a sensitivity of 99.0% (mpMRI) and 97.1% (bpMRI). McNemar’s chi-squared test of 4.2 revealed significant differences between mpMRI and bpMRI (*p* < 0.001). Specificity was 47.5% (mpMRI) and 61.2% (bpMRI), which was also significantly different between tests (McNemar’s chi-squared test of 33.0, *p* < 0.001). The presence of a csPCa in histology was considered the gold standard. Additional values for PPV and NPV are summarized in Table 2.

**Table 2:** Accuracy of multiparametric MRI (mpMRI) and biparametric MRI (bpMRI) with histology as gold standard

PI-RADSv2.1 assessment	mpMRI	bpMRI	
Sensitivity (CI)	99.0% (97.2%–99.8%)	97.1% (94.5–98.7%)	McNemar’s test 4.2, *p* < 0.001
Specificity (CI)	47.5% (41.2–53.8%)	61.2% (54.9–67.2%)	McNemar’s test 33.0, *p* < 0.001
PPV (CI)	69.5% (66.9–71.9%)	75.1% (72.1–77.9%)	WGS 29.8, *p* < 0.001
NPV (CI)	97.6% (92.9–99.2%)	94.6% (90.0–97.1%)	WGS 3.4, n.s

*PPV* positive predictive value; *NPV* negative predictive value; *CI* confidence interval; *WGS* weighted generalized score test statistic

## Discussion

Multiparametric MRI has undoubtedly gained momentum as a diagnostic tool to detect prostate cancer and is currently recognized as the best imaging method for assessing primary prostate cancer. While the current PI-RADSv2.1 protocol demands the use of contrast-enhanced sequences, several recent investigations have evaluated the use of bpMRI without DCE for several indications [15, 16].

Here, we challenged the need for mpMRI in a patient cohort with a clinical suspicion of prostate cancer that was scheduled for an MRI before histopathology from RA-TB was obtained. MRI examinations were independently read as bpMRI or mpMRI in separate sessions. In our study, all mpMRI scans were performed on 3 T scanners, adhering to the PI-RADSv2.1 protocol, undertaken by trained prostate-MRI technologists.

In this retrospective study in patients with high-risk for PCa comparing mpMRI including contrast agent and bpMRI, a sensitivity/specificity/PPV/NPV for mpMRI of 99.0%/47.5%/69.5%/97.6% and a sensitivity/specificity/PPV/NPV of 97.1%/61.2%/75.1%/94.6% for bpMRI were found.

The high imaging standards and experienced readers helped limit the proportion of “uncertain” (PI-RADS 3) diagnoses. PI-RADS 3 was present in 16.9% for mpMRI in our study, versus 28% and 21%, in the promis and precision trail [17, 18], respectively. These multicenter studies were performed on 1.5 T or 1.5 and 3 T scanners, respectively, resulting in more variability in acquisition parameters and multiple readers which might explain the slight difference in PI-RADS 3 findings.

The number of PI-RADS 3 lesions detected that were positive on biopsy was 32.6%, 9.7% of those had csPCa. This is in line with other studies [19–21] demonstrating the overall low rate of csPCa among the PI-RADS 3 lesions, even in a study collective with clinical high prevalence of PCa.

The number of PI-RADS 3 findings with positive DCE leading to an upgrade to PI-RADS 4 with subsequent diagnosis of a csPCa was limited in this cohort (*n* = 6) compared to 36 cases upgraded to PI-RADS 4 due to positive DCE with no evidence of cancer upon biopsy. This number is relatively low compared to other studies, which might be due to the highly selected cohort of patients scheduled for biopsy. A recent study by Sherrer et al. [22] also found a relative low number of DCE-positive findings that would have been missed on bpMRI.

Robot-assisted transperineal fusion prostate biopsy was used in all cases for biopsy resulting in a very standardized biopsy procedure that is not commonly used in comparable studies [23, 24]. The number of positive cores as reported by the pathologists was used. We did not include the core length or the percentage of infiltration in our analysis [25].

This paper makes contributions to existing literature with controversy regarding the use of a bpMRI approach in men with suspicion of PCa. While some publications demonstrate the necessity for DCE in line with current recommendations of the PI-RADS committee [26, 27], a recent meta-analysis concludes that bpMRI is feasible csPCa detection [15]. The need for defined indications, however, is emphasized in this evaluation. They conclude that the broad variability in sensitivity and specificity for detection of csPCa in the analyzed studies might primarily be influenced by reader experience and the disease prevalence in the patients included.

Our study provides evidence that the bpMRI pathway demonstrates similar sensitivity and better specificity compared to the mpMRT pathway in men with high clinical suspicion for PCa. Sensitivity/specificity was 99.0/47.5% for mpMRI and 97.1/60.2% for bpMRI in our study regarding detection of csPCa. A wide range in sensitivity (45–95%) and specificity (45–100%) for bpMRI are reported in a recent meta-analysis for the detection of PCa [28], as well as for csPCa [29] with sensitivity between 44 and 100%) and specificity (15–97%). With high sensitivity values and fair specificity values, our results reflect the preselected cohort with a disease prevalence of 59.9%.

Sensitivity and NPV are high in mpMRI in our study which can be explained by the high-risk population for PCa. Due to the high clinical suspicion for PCa, all patients underwent biopsy irrespective of the MRI findings as mentioned in the manuscript. Moreover, very high sensitivity and NPV are not uncommon for high-risk populations as demonstrated in several studies [29–33]. Thus, Cuocolo et al. emphasize the need for a standardized imaging protocol and prospective studies for validation of bpMRI as current studies are very heterogenous in design [29]. The PI-RADS steering committee has also recognized the increasing demand for prostate MRI in general and recently discussed possible applications for bpMRI considering high-quality imaging, expert interpretation quality and clinical risk stratification [16]. The need for prospective studies with biopsy decisions made according to MRI without DCE and definite clinical and operational benefits is again highlighted.

This study has several limitations. First, this is a retrospective single center study with a preselected clinical population scheduled for biopsy with no randomization. Second, RA-GB was planned due to clinical suspicious of PCa, regardless of mpMRI results, including PI-RADS 2 scores. This may be considered as potential bias inflating the PCa detection rates in off-target-biopsy. Another potential limitation of this study design is a possible change of the index lesion when bpMRI is applied compared to mpMRI as no update to PI-RADS 4 in PZ lesions with positive DCE is applicable. This was not the case in the cohort of investigation as no competing lesion was present in these cases and PI-RADS 3 lesions were also considered for biopsy. However, this will be of relevance in a prospective randomized trial with limitation of biopsy to ≥ PI-RADS 4 lesions. The examinations in this study were acquired before the PI-RADS v2.1 update in 2019, however, the MRI examination protocol fully met the technical requirements proposed there. We did not evaluate the family history on prostate cancer for this study which can limit further evaluation of the investigated population. Regarding histological evaluation of the biopsies, cancer core length and percentage of infiltration were not assessed in this study.

In summary, bpMRI demonstrated better specificity and positive predictive value while mpMRI showed slightly better sensitivity and negative predictive value in a real-world population with high risk for prostate cancer scheduled for RA-TB.

## Conclusion

In patients with a suspected PCa and elevated PSA scheduled for biopsy, mpMRI demonstrated slightly better sensitivity while specificity was superior in bpMRI for the detection of csPCa in a cohort of high-risk patients for PCa. We conclude that bpMRI is sufficient for planning and performance of targeted biopsy in patients with suspected PCa in biopsy naïve patients undergoing first RA-TB biopsy. These findings need to be confirmed in prospective, randomized studies before mpMRI can be recommended in selected cases.

## Data Availability

All relevant data are included in the manuscript.
